# Study of narrow negative magnetoresistance effect in ultra-high mobility GaAs/AlGaAs 2DES under microwave photo-excitation

**DOI:** 10.1038/s41598-019-57331-9

**Published:** 2020-01-21

**Authors:** R. L. Samaraweera, B. Gunawardana, T. R. Nanayakkara, R. C. Munasinghe, A. Kriisa, C. Reichl, W. Wegscheider, R. G. Mani

**Affiliations:** 1 Department of Physics and Astronomy, Georgia State University, Atlanta, 30303 Georgia USA; 2 Laboratorium für Festkörperphysik, ETH Zürich, CH-8093 Zürich, Switzerland; 3 Department of Science and Technology, Uva Wellassa University, Badulla, 90000 Sri Lanka

**Keywords:** Physics, Condensed-matter physics

## Abstract

The microwave-induced change in the narrow negative magnetoresistance effect that appears around zero magnetic field in high mobility GaAs/AlGaAs 2DES (≈10^7^ cm^2^/*Vs*) is experimentally examined as a function of incident microwave power at a fixed bath temperature. The experimental results indicate that the narrow negative magnetoresistance effect exhibits substantially increased broadening with increasing microwave intensity. These magnetoresistance data were subjected to lineshape fits to extract possible variation of characteristic lengths with microwave intensity; the results suggest that characteristic lengths decrease by up to 50% upon increasing microwave power up to about 8 mW. We also examine the change in effective electron temperature, *T*_*e*_, due to the photo-excitation in the absence of a magnetic field. Combining these results suggests a correlation between electron heating and the observed change in the fit extracted characteristic lengths.

## Introduction

Advances in molecular beam epitaxy have facilitated the growth of high mobility GaAs/AlGaAs heterostructures with ever improving material quality which show interesting novel physical phenomena including the radiation-induced zero resistance states and associated magneto-resistance oscillations at low magnetic fields and liquid helium temperatures^1–32^. By now, a number of experimental^1–32^ and theoretical^33–50^ studies have been carried out on the photo-excited transport in low dimensional systems. In addition to the photo-excited effects, the dark magneto-transport properties including positive magnetoresistance^51,52^, giant negative magnetoresistance^53–61^, and narrow negative magnetoresistance effect near zero field have also attracted recent experimental attention. So far as the larger negative giant magneto-resistance is concerned, recent studies have shown remarkable features such as size dependence, tunability with supplemental dc-current, and coexistence with- and separability from- radiation induced magnetoresistance oscillations^59–65^. One emerging theory suggests that some of the observed features of the larger negative giant magnetoresistance effect could be a signature of a viscous electron liquid in the low magnetic field quasi ballistic transport regime. Yet, the exact origin of the narrow negative magnetoresistance effect in ultra-high mobility GaAs/AlGaAs 2DES specimens is still an open topic for investigation both from the experimental^56,58^ and theoretical perspectives.

Thus, we examine here by experiment, the influence of microwave power on the narrow-negative magnetoresistance effect in the high quality GaAs/AlGaAs 2D electron system. A brief report on our early work appears at ref. ^63^. Here, we present data as a function of several parameters, and subject the observed narrow negative magnetoresistance data to an empirical fit in order to extract the characteristic lengths. Concurrently, we extracted the change in the electron temperature due to possible microwave heating of the 2DES from the zero magnetic field resistance data, i.e., ${R}_{xx}$ at $B=0$ Tesla. The results suggest a 50% drop in the narrow magnetoresistance fit extracted characteristic length over a change in source microwave power of 8 mW. The observed reduction in the characteristic lengths under microwave excitation are attributed to electron heating and dephasing by microwave excitation.

## Results

**Figure 1:**
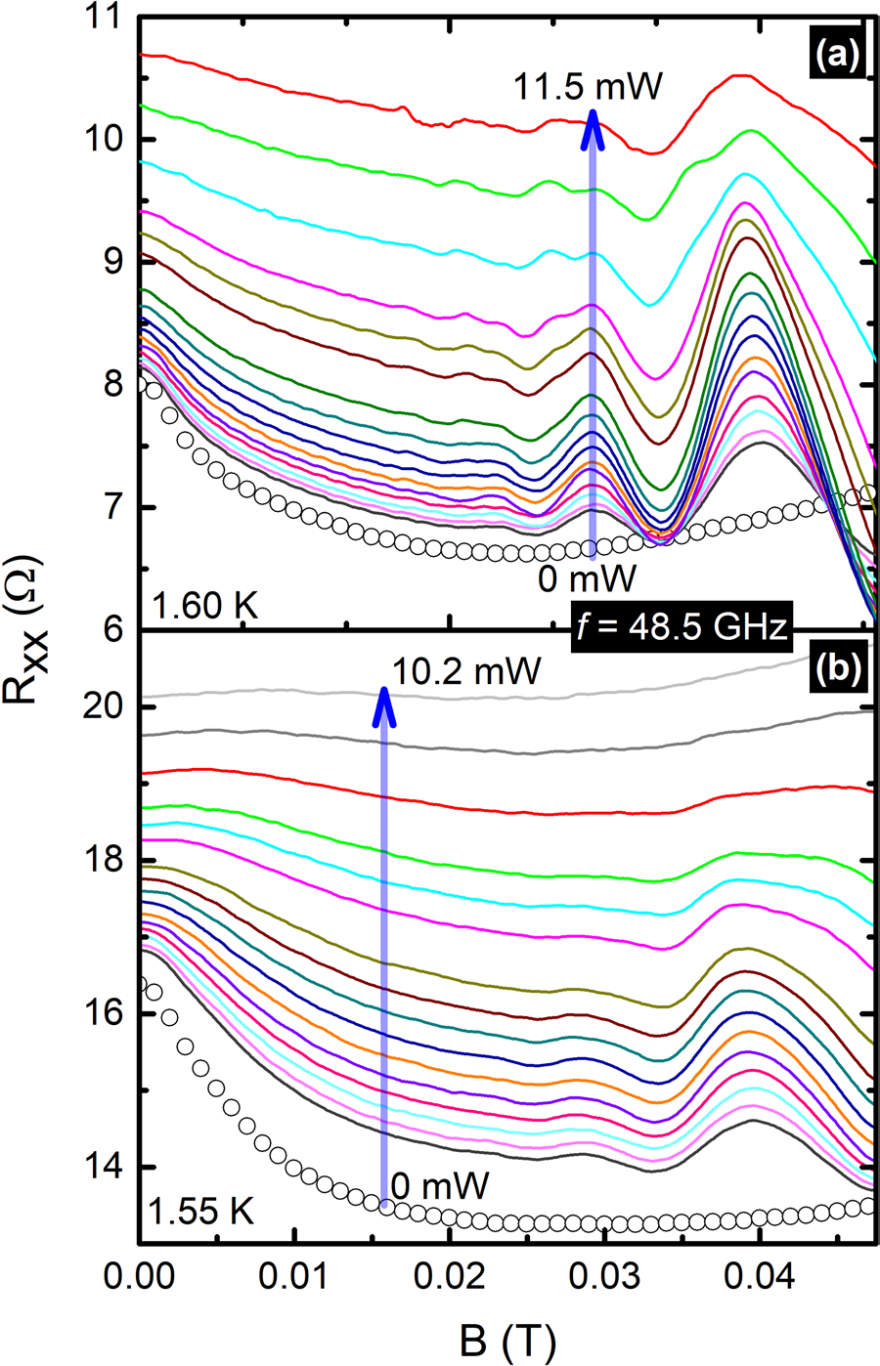
This figure shows the experimental data i.e. ${R}_{xx}$ vs B, over the magnetic field range of $0\le B\le 0.045$ Tesla. Photo-excited ${R}_{xx}$ data are shown at microwave frequency, $f=48.5\,{\rm{GHz}}$ for (**a**) the higher mobility sample measured at 1.60 K and (**b**) the lower mobility sample at 1.55 K bath temperature. The vertical arrows indicate the direction of increasing power from 0 mW to ≈12 mW. Lines with symbols in (**a** and **b**) exhibit the corresponding dark curves.

Figure 1 exhibits magnetoresistance data under dark and microwave photoexcited conditions with frequency $f=48.5\,{\rm{GHz}}$, with the microwave power *P* as the parameter. One can observe typical magnetoresistance features observed in the GaAs/AlGaAs 2DES, including non-oscillatory magneto-resistance effects such as the narrow negative-magneto-resistance effect, that span over $0\le B\le 0.025$ Tesla and oscillatory radiation-induced magnetoresistance oscillations at $B\ge 0.02$ Tesla. Figure 1(a) exhibit the data for a higher mobility sample with carrier mobility ${\mu }_{B}=1.18\times {10}^{7}\,c{m}^{2}/Vs$, and Fig. 1(b) exhibits that of lower mobility sample with ${\mu }_{C}=0.66\times {10}^{7}\,c{m}^{2}/Vs$. Both samples show that the ${R}_{xx}$ at $B=0$ increases with increasing microwave power. Also, the full-width at half maximum (FWHM) of the narrow negative-magneto-resistance peak increases as a function of microwave power. Here, one can observe relatively weak radiation-induced magnetoresistance oscillations in the lower mobility sample (i.e., Fig. 1(b)) and stronger oscillations in the higher mobility sample (i.e. Fig. 1(a)). The data show an initial growth in radiation-induced magnetoresistance oscillation amplitude with microwave power followed by a decrease again upon further increasing the applied microwave power. The radiation-induced magnetoresistance oscillations observed in the lower mobility sample (Fig. 1(b)) completely disappears at elevated microwave power i.e. at about *P* = 8.5 mW. Also, the negative-magneto-resistance effect quenches faster in the lower mobility specimen (Fig. 1(b)) than the higher mobility specimen (Fig. 1(a)). Figure 1(b) indicates that the negative-magneto-resistance effect almost disappears by *P* = 10.2 mW, whereas in higher mobility specimen it is not completely quenched even at *P* = 11.5 mW. Possible explanations for these observations include: (i) the effective microwave linear polarization angle at the sample site may be different for two samples, (ii) the inelastic scattering rates at a given condition may be different for the two samples due to their differing mobility. To extract further information into the effect of microwave excitation, the data were fit to:

1 $${\rho }_{(B)}={\rho }_{(0)}-\frac{{e}^{2}{\rho }^{2}}{2{\pi }^{2}\hslash }[\psi (\frac{1}{2}+\frac{{B}_{i}}{B})+\,ln\,\frac{B}{{B}_{i}}]$$

Here, $\psi$ is the digamma function, $\rho$ resistivity, and

2 $${B}_{i}=\frac{\hslash }{4e{l}_{i}^{2}}$$

Although this is a weak localization line-shape^64^, we do not interpret the observed effect as canonical weak localization. The observation of weak localization effect requires that the inelastic length, ${l}_{i}$, exceeds the elastic length, ${l}_{e}$, i.e., ${l}_{i} > {l}_{e}$^66^. However, the extracted inelastic lengths, ${l}_{i}$, for these samples are in the range of few micrometers, and they are much smaller than the, elastic length, ${l}_{e}$, which is about 100 *μm* at *T* = 1.5 *K*. Therefore, we are just using this line-shape to track observable changes under microwave photoexcitation, given the absence of any other candidate fitting functions.

**Figure 2:**
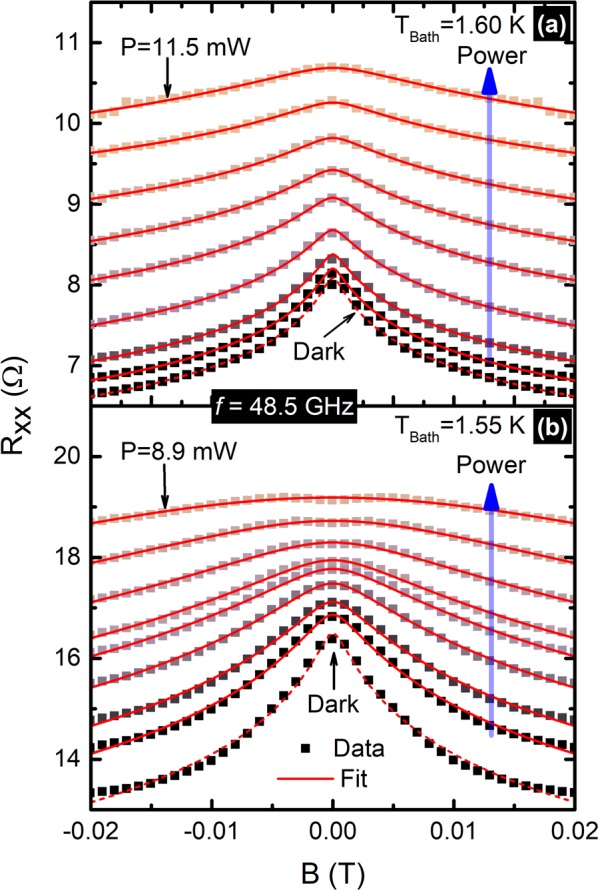
This figure shows the non-oscillatory part of the photo-excited magneto-resistance data that spans over −$0.02\le B\le 0.02$ Tesla. The vertical arrows indicate the direction of increasing microwave power from 0 mW to 12 mW, under photo-excitation of frequency, $f=48.5\,{\rm{GHz}}$. This panel shows selected data (squares) and corresponding fits (lines) for (**a**) the higher mobility sample measured at 1.60 K and (**b**) the lower mobility sample at 1.55 K bath temperature. Dashed lines in (**a** and **b**) exhibit the fits of dark magneto-resistance data to Eq. (1).

**Figure 3:**
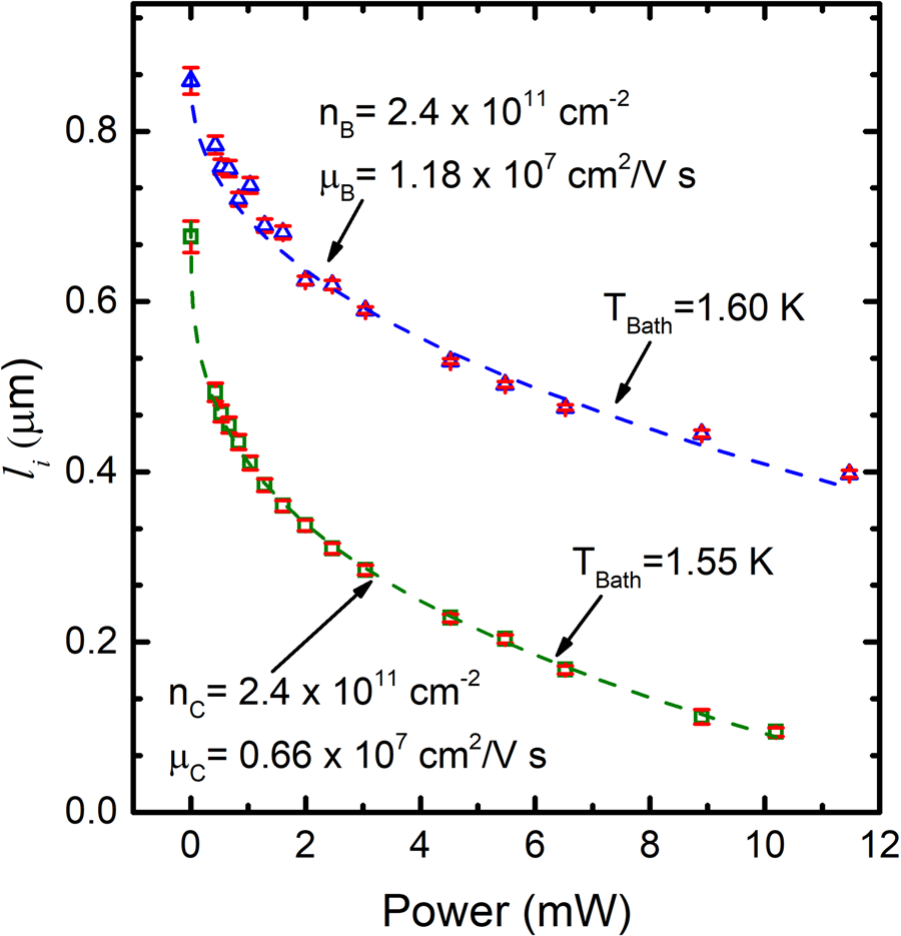
This figure exhibits the microwave power dependence of the fit extracted ${l}_{i}$, see text, for the higher mobility sample at 1.60 K and the lower mobility sample at 1.55 K, as labeled in the graph. Dashed-lines are guides to the eye.

We fit the observed small and narrow negative magnetoresistance effect to Eq. (1). The only parameter that may be extracted from line-shape fits of the data is ${l}_{i}$. We call it a characteristic length while keeping in mind that it is referred to as the inelastic length in weak localization. The Fig. 2 exhibits the fit (solid line) of power dependent narrow negative-magneto-resistance data (squares) to Eq. (1). As shown in the Fig. 2, the experimental data, that span over the range −$0.02\le B\le 0.02$ Tesla are well described by the fits. Figure 3 summaries the fit extracted ${l}_{i}$. In both samples, ${l}_{i}$ decreases monotonically with increasing microwave power. Also, ${l}_{i}$ is smaller in lower mobility sample than the higher mobility sample. Here, it turns out that the influence of nominal power on ${l}_{i}$ is more pronounced in lower mobility sample than the higher mobility sample. For example, ≈50% drop in ${l}_{i}$ occurs by about *P* = 8.5 mW for sample in Fig. 1(a), while it takes place by about *P* = 2 mW in sample in Fig. 1(b). This feature could also be due to the difference in effective power experienced by the two samples due to differences in the microwave polarization angles at the sample site. Next, we further examined the influence of microwave power on the narrow negative-magneto-resistance effect aiming to uncover a possible mechanism that explains the observations, including quenching of the ${l}_{i}$ at high power.

**Figure 4:**
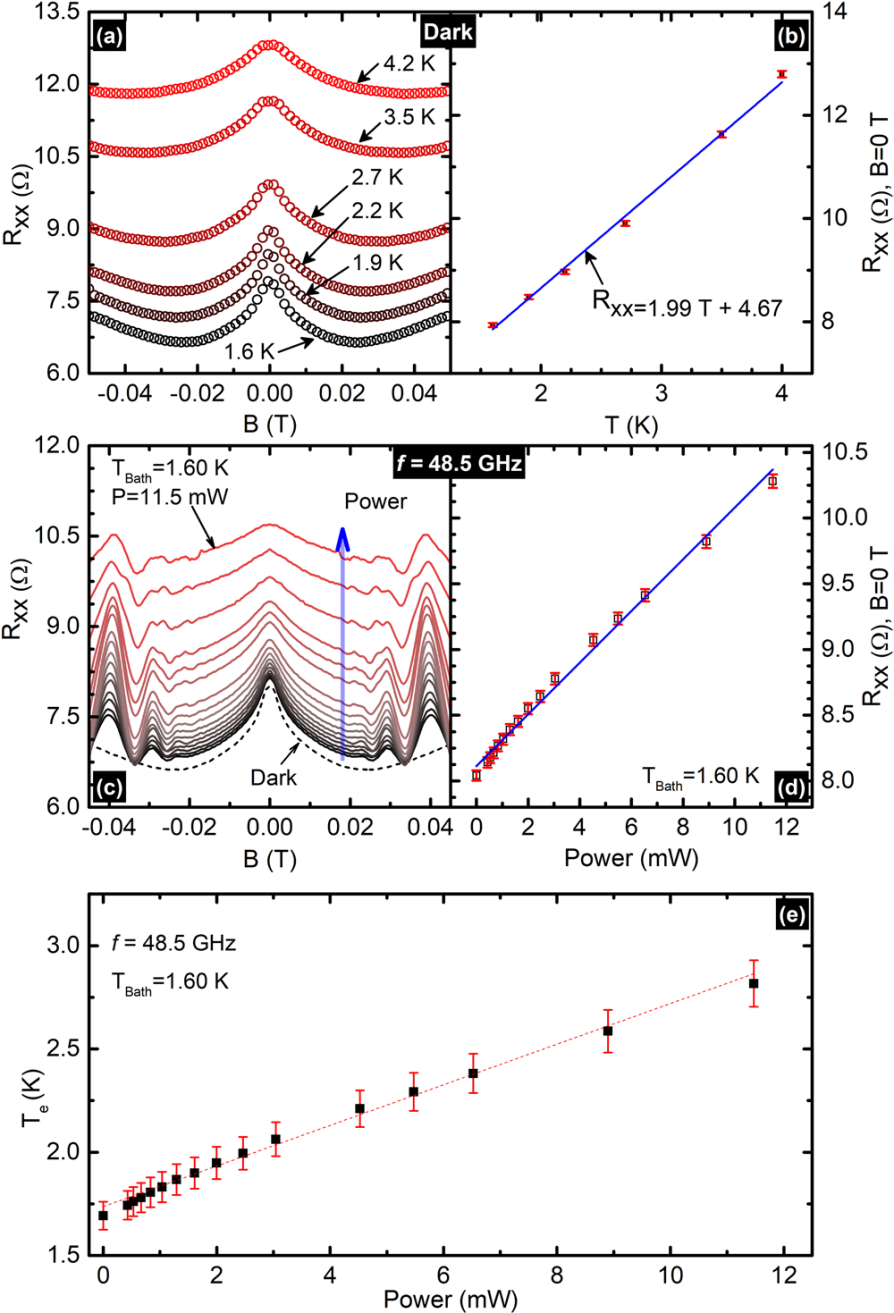
(**a**) This figure shows dark magnetoresistance data i.e. ${R}_{xx}$ vs $B$ for −$0.05\le B\le 0.05$ Tesla of the higher mobility sample at different temperatures from 1.60 K to 4.20 K. (**b**) Exhibits the ${R}_{xx}$ at *B* = 0 Tesla vs. the bath temperature, $T$, the solid line represent the best linear fit to the data. (**c**) Photo-excited ${R}_{xx}$ data of the higher mobility sample at various powers are shown at microwave frequency, *f* = 48.5 GHz. (**d**) Zero field resistance i.e. ${R}_{xx}$ at *B* = 0 Tesla vs. the microwave power $P$, the solid line represent the best linear fit to the data (**e**) this panel shows the calculated electron temperature ${T}_{e}$ vs $P$ for the higher mobility sample.

Theory suggests, and previous experimental reports have confirmed, that microwave radiation absorbed by the 2DES leads to an increase in the electron temperature above the lattice temperature. We utilize the resistance data at $B=0$ Tesla to estimate the electron temperature in the vicinity of null magnetic field. In this approach, the up-shift in the zero-field resistance under microwave excitation was used to estimate the electron temperature by comparing with the up-shift in the resistance with the bath temperature in the absence of microwave photoexcitation. Figure 4(a–e) exhibit the main steps involved in the determination of the effective electron temperature using zero field resistance data. Data and analysis are shown here for the high mobility specimen only to illustrate the procedure. Figure 4(a) shows the effect of bath temperature, $T$, between $1.6\,K < T < 4.2\,K$, on the magnetoresistance, ${R}_{xx}$ under dark conditions. Clearly, the narrow negative magnetoresistance effect is sensitive to temperature. We used this data to extract ${R}_{xx}$ at *B* = 0 Tesla and Fig. 4(b) shows that the extracted ${R}_{xx}$ at *B* = 0 increases monotonically with increasing bath temperature. Similarly, we suspected that the absorption of microwave radiation may increase the effective electron temperature above the lattice temperature and consequently lead to an increase in the zero-field resistance. Thus, we used the temperature dependent zero field resistance data (Fig. 4(b)) as a calibration to determine the effective electron temperature due to the absorbed microwave radiation. The Fig. 4(c) shows typical microwave induced magnetoresistivity data in the field range of −$0.045 < B < 0.045$ Tesla, under the excitation of frequency, $f=48.5\,{\rm{GHz}}$ at various microwave power. Figure 4(d) shows the extracted ${R}_{xx}$ at *B* = 0 vs the microwave power increases monotonically with increasing power. We used the slope and intercept interpreted from the linear fits to the data in Fig. 4(b) to calculate the effective temperature increment against the applied microwave power. Figure 4(e) exhibit the effective electron temperature vs the microwave power of the higher mobility sample at a bath temperature of 1.60 K. Here the rate of change in electron temperature is about 0.098 K/mW, and that for lower mobility sample is 0.183 K/mW. The results suggest that electron heating in the lower mobility sample is greater than that of the higher mobility sample.

**Figure 5:**
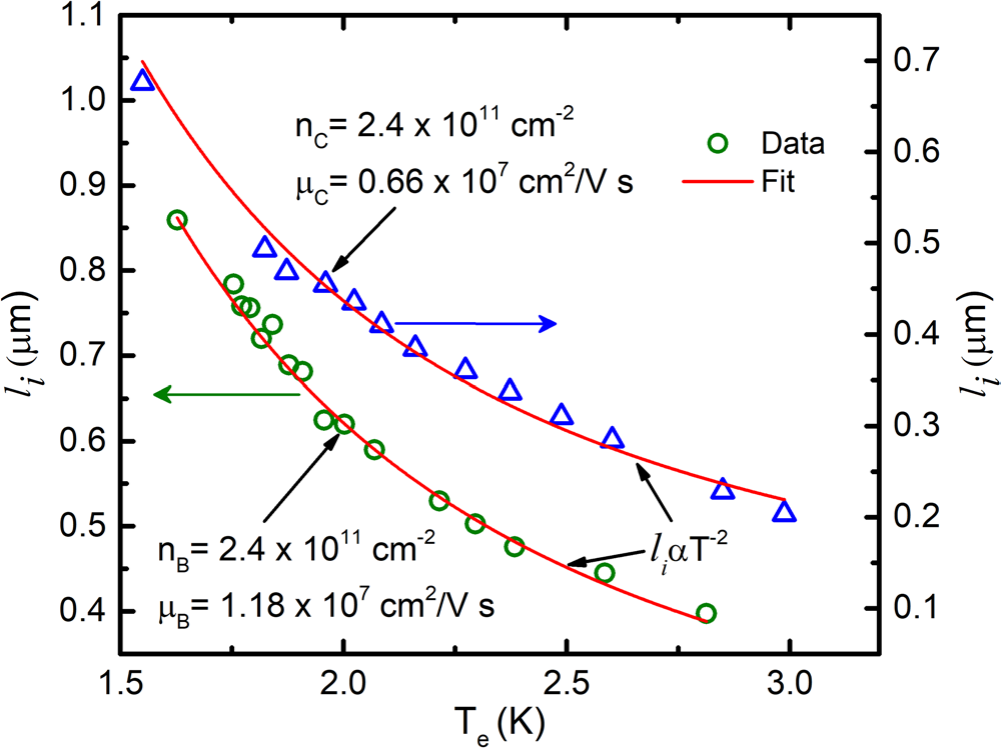
This figure shows the ${l}_{i}$ vs ${T}_{e}$ for the data shown in Fig. 3, see text. Note that ${T}_{e}$ is the temperature calculated using zero field resistance. Solid lines represents ${T}^{-2}$ law fit to the ${l}_{i}$ data for the higher mobility sample (Left ordinate) and the lower mobility sample (Right ordinate)).

Figure 5 exhibits the ${l}_{i}$ vs electron temperature, ${T}_{e}$ that is determined using the zero-field resistance data as described above. For both samples, the ${l}_{i}$ data follows the ${T}^{-2}$ curve (solid-lines in Fig. 5). If this were weak localization, one would interpret the ${l}_{i}\propto {T}^{-2}$ as suggestive of electron-electron type inelastic scattering. One can understand the influence of microwave radiation on the narrow negative magneto-resistance effect as electron heating due to the absorption of microwave radiation by the 2DES. Absorption of microwave results in increasing the electron temperature that eventually increases the electron-electron scatterings leading to quenching of ${l}_{i}$. Simply put, it looks as though microwave photo-excitation reduces ${l}_{i}$ at a fixed lattice temperature near null magnetic field.

## Discussion

This work aimed to explore the influence of microwave radiation on the narrow negative magneto-resistance effect at $B=0$ Tesla in ultra-high mobility GaAs/AlGaAs 2DES, at a fixed bath temperature. The experimental data were fit using the Hikami 2D WL theory^64^, neglecting the spin orbit scattering term, and also electron-electron interaction effects. Our previous reports reveal the absence of electron-electron interaction effect in these specimens^57,65^. In the presence of spin-orbit interaction one would expect a positive magnetoresistance in these types of device structures, rather than a negative magnetoresistance^51,66^. However, the specimens studied here do not show such positive magnetoresistance feature in the vicinity of zero magnetic field. Therefore, we can neglect the spin-orbit scattering term as well in the fitting equation.

The disappearance of narrow negative magnetoresistance under high-power microwave photo-excitation can occur due to either heating effect or dynamic suppression of backscattering by the incident microwaves^40^. Theoretical predictions and experimental observations suggest that absorption of energy from microwave radiation results in increasing the 2D electron’s temperature above the lattice temperature. Even though the energy absorption rate is small in high-mobility 2DES at liquid helium temperatures, electron heating is still significant, because at the same time, the energy dissipation rate to the lattice thorough electron-phonon scattering is also small.

In order to understand the electron heating effect in the vicinity of zero magnetic field, we evaluated the effective electron temperature using zero field resistance change due to the microwave excitation. Our results show that the effective electron temperature calculated using zero field resistance is greater than previously reported values that were calculated using amplitude damping of SdH oscillations^20^. The observed differences can be attributed to the strong dependence of the microwave energy absorption rates by 2DES on the B-field, as theoretically predicted by Lei *et al*.^21,40^. Further, both theoretical and experimental studies have examined the influence of MW polarization on the oscillatory magnetoresistance effect as well as the electron heating in GaAs/AlGaAs 2DES^23,47,67,68^. For example, it is shown that the absorption rate and the electron temperature are independent of the polarization direction, especially in the high magnetic fields^67^. However, we need further experiments and theoretical investigations to explain the exact polarization dependency of the narrow-negative magnetoresistance effect that are observed near-zero field.

We have previously reported the effect of bath temperature on the inelastic scattering length^65^, where the measurements were taken under dark conditions, i.e. without microwaves. A comparison of present results with the previous observations reveals that the influence of microwave power on the fit extracted ${l}_{i}$ is stronger than that of the bath temperature. That is, the rate of change in ${l}_{i}$ is more pronounced in the presence of microwave radiation. For example, a 50% change in ${l}_{i}$ occurs when the bath temperature is increased up to about 4.5 K. However, in the presence of microwaves, 50% drop in ${l}_{i}$ takes place when the calculated ${T}_{e}$ is only about 2.8 K. This indicates the need for further investigations on other possible mechanisms that destroy the phase coherence of carriers in the presence of microwave excitation such as the effect of radio-frequency (rf) electric field on the quantum-mechanical correction to the conductivity/resistivity^66^. It is thought that the rf electric field should disrupt the phase of carriers wave function. Thus, microwave radiation could be more effective in decreasing the ${l}_{i}$ in comparison to simply heating the specimen by increasing the bath temperature.

Thus, the observed microwave power dependence in the narrow-negative magneto-resistance feature can be interpreted as a consequence of electron heating due to the energy absorbed from the microwave radiation. The energy absorbed from the radiation field equilibrates the electronic system above the bath temperature thus increasing the electron-electron scattering rate. This mechanism is supported by the fit extracted ${l}_{i}$, which decreases with increasing microwave power.

## Conclusion

In summary, we observed a significant change in the narrow negative-magneto-resistance effect under microwave irradiation as a function of the power at a fixed bath temperature, and fit these data with an empirical lineshape to extract characteristic lengths. The change in the narrow negative magnetoresistance lineshape under microwave photoexcitation is expected to originate as a result of excess electron heating well above the lattice temperature due to the absorption of energy from the radiation field near null magnetic field. Thus, we measured the effective electron temperature change due to the microwave photoexcitation using the dark, zero-field resistance data as a thermometer. With this approach, the microwave power could be translated to an electron temperature, and the extracted characteristic lengths could be plotted versus the effective electron temperature at each microwave power. It appears that the energy absorbed from the radiation field thermalizes the 2DES system above the bath temperature and increases the effective electron temperature, thus increasing the electron-electron scattering rate. As a result of increasing scattering rates, the ${l}_{i}$ decreases and the associated narrow negative-magneto-resistance feature in 2DES disappears with increased photo-excitation at a fixed bath temperature.

## Methods

High mobility MBE grown GaAs/AlGaAs heterostructures were patterned into Hall bars by photolithography. 2D electron mobility *μ* and the density *n* of the two samples at ≈1.6 K were ${\mu }_{B}=1.18\times {10}^{7}\,c{m}^{2}/Vs$, ${n}_{B}=2.4\times {10}^{11}\,c{m}^{-2}$ and ${\mu }_{C}=0.66\times {10}^{7}\,c{m}^{2}/Vs$ and ${n}_{C}=2.4\times {10}^{11}\,c{m}^{-2}$ respectively. The elastic scattering length ${l}_{e}$ of the high-mobility specimens were calculated at the lowest temperature using the expression ${\sigma }_{0}=n{e}^{2}{\tau }_{e}/{m}^{\ast }={e}^{2}{k}_{F}{l}_{e}/h$, and ${k}_{F}=\sqrt{2\pi n}$, where ${\sigma }_{0}$-zero field conductivity, $n$-electron density, $e$-elementary charge, ${\tau }_{e}$-elastic scattering time, m*-electron effective mass, ${k}_{F}$-Fermi wave vector and $h$-the Plank’s constant^66,69^. The Four terminal electrical measurements were carried out on the Hall bars using low-frequency lock-in based techniques with the sample mounted at the end of a cylindrical waveguide, within a variable temperature insert, inside a superconducting solenoid in the $B\perp I$ configuration. Sample was illuminated using *f* = 48.5 GHz microwaves through the cylindrical waveguide. The samples were immersed in liquid helium, and temperature control was realized by controlling the vapor pressure of liquid helium. In this experiment, the incident microwave power $P$ was varied as the parameter by means of a power amplifier and attenuator system. Typically, magnetic field (*B*) sweeps of the lock-in detected diagonal voltage ${V}_{xx}$ were collected at a fixed microwave power at ≈1.60 K, in order to determine magnetoresistance, ${R}_{xx}={V}_{xx}/{I}_{ac}$.
